# Supplementary material for: Are carbon emissions trading and green financial instruments synergistic? -Comprehensive quantitative research based on content analysis
Source: PLoS One. 2024 Mar 7;19(3):e0298601. doi: 10.1371/journal.pone.0298601 (PMC10919601; doi:10.1371/journal.pone.0298601)
Supplement: S1 Data — (ZIP) [file pone.0298601.s001.zip › Data/地级市Z_F工作报告.zip!/║■─╧/╗│╗»╩╨/└┤╫╘╣1⁄2╓┌║┼í╛┬φ┐╦╩2╛▌═°í┐_2015.docx]

即将过去的一年，我们在中共怀化市委的坚强领导下，认真贯彻落实中央和省委、省政府决策部署，团结拼搏，攻坚克难，经济社会保持平稳发展。预计全年实现生产总值1180 亿元， 增长5.1%； 完成全社会固定资产投资870 亿元，增长8.5%；完成规模工业增加值300 亿元，增长7%；实现社会消费品零售总额407 亿元， 增长12.7%； 完成公共财政收入100.1 亿元、负增长9.5%，地方财政收入69.5 亿元、负增长9.5%； 城乡居民收入分别达到16620 元、6660 元，增长9.5%和11%。

——主动应对经济持续下行新压力。坚持问题导向，及时研判形势，实施系列保增长、稳增长、促增长措施，经济下滑势头在下半年得到一定遏制，发展质量有所提升。新兴工业企业加快成长。完成工业投资260 亿元，实施重点工业项目58 个，托口水电站等项目建成投产。开工建设标准化厂房115.5 万平方米，建成50万平方米。完成高新技术产业增加值52.5 亿元，增长8%。实施重点减排项目154 个，淘汰落后水泥生产线7条，关闭落后产能企业6 家。万元规模工业增加值能耗下降12%。农业品牌培育取得突破。深入推进十大农产品基地建设，创建农业标准化核心示范园区114 个，建设种植业标准化园区401 万亩。溆浦超级稻百亩示范片创造亩产1026.7 公斤新纪录。新晃“侗藏红米”入列“中国重要农业文化遗产”名录。金珠米业“湘怀金珠”获中国驰名商标。碣滩茶、麻阳白鹅获国家地理标志证明商标，总量增加到13 件，居全省第一。新增市级以上农业产业化龙头企业22 家。农作物耕作机械化水平达到39.6%。第三产业升级步伐加快。实施重点商贸物流项目38 个，狮子岩物流园区跻身“全国优秀物流园区”。洪江古商城获评国家4A 级景区，溆浦思蒙获批国家级湿地公园。粟裕同志纪念馆、通道转兵纪念馆改造竣工并对外开放。成功举办纪念通道转兵80 周年暨湖南红色旅游文化节· 怀化第四届“三古”文化旅游节。全年接待国内外游客增长15%，旅游总收入增长16%。完成房地产开发投资110亿元，销售商品房290.5 万平方米，增长6.5%。会展经济来势看好，市汽博会、房交会累计成交8.4 亿元。

——扎实推进基础设施建设新突破。实施重点建设项目270 个，完成投资394 亿元。长昆客运专线怀化段建成通车，怀化正式进入“高铁时代”。娄怀高速公路将于12 月31 日全线贯通，成为怀化东进开放的又一条高速通道；绕城高速公路将同日通车，怀化成为我省除省城外第一个拥有绕城高速的地级市。怀邵衡铁路、武靖高速公路等项目开工建设。芷江机场跑道完成路基工程，新开深圳、成都两条航线。改造完成G209 怀化至麻阳、G320 芷江至新晃国省干线100 公里。新增农村通畅工程141 个村、612 公里，通畅率提高到97%。提质改造中心城区背街小巷110条，竣工95条。氵舞阳大道一期、平安路二期、市第二污水处理厂等项目基本建成。氵舞水河综合治理、城区四个高速公路主入口拓改、中坡景观工程建设等项目顺利推进，建成污水管网440 公里。中心城区建成区面积达到64 平方公里，常住人口突破60 万人，全市城镇化率达到42%，提高1.6 个百分点。实施农田水利重点项目48 个，除险加固病险水库114 座，新建和加固堤防112 公里，建设小型农田水利工程4 万多处，新增和改善有效灌溉面积40 万亩。解决29 万农村人口饮水不安全问题。

——切实增强全面深化改革新活力。市本级行政审批事项减少234 项，保留96 项, 精减71%。下放怀化工业园市级经济管理权限77 项，靖州太阳坪简政放权与便民服务试点形成可复制经验并在全市推广。国有企业改制及遗留问题处理步伐加快。恒光科技在新三板成功挂牌，实现本土中小企业新三板市场融资零突破。长沙银行怀化分行开业运营。市级投融资平台融资54.84 亿元，全市中小微企业贷款余额289 亿元、增长14%。推行工商登记制度改革，全市市场主体达到17.9 万户、增长20%，注册资本金增长145%。麻阳农村产权制度改革试点、溆浦土地承包经营权确权颁证改革试点等取得阶段性成果。全口径预算管理、“营改增”等改革顺利推进。深化开放合作，全年引进产业项目195 个，实际到位市外境内资金423 亿元，增长35%。到位外资和进出口总额双双突破1 亿美元，分别增长15%、25.9%。怀化铁路口岸获省政府批准建设。芷江抗战受降旧址和纪念馆、飞虎队纪念馆入列首批国家级抗战纪念设施、遗址名录，通道、靖州分别获批全省文化旅游产业重点县、农副产品加工产业重点县。推动科技创新，与湘潭大学签署战略合作协议，通道万佛山农业科技园获批省级农业科技园区，补天药业科技创新项目获省科技进步一等奖。省级企业技术中心增加到8 家，院士专家站达到2 家，科技项目进入国家计划12 个、省级计划128 个，授权专利354 件。

——全力强化民生持续改善新保障。尽管今年政府极为困难，财力十分紧张，但在保障民生支出上做到了应保尽保。财政用于民生支出增长20%， 占财政总支出的74.8%。全面完成省考核的15 类29 项为民办实事任务。新增创业主体1.4 万个、城镇就业3.7 万人。完成农民技能培训12.7 万人次，新增农村劳动力转移就业5.4 万人。新（ 改）建农村合格学校102 所、农村公办幼儿园23 所、教师周转房2636 套，改造中小学C、D 级危房17 万平方米，铁二中、铁一中、顺天路中学改扩建进展顺利。怀化医专成功申建湖南医药学院，成为武陵山片区第一所独立建制的公办医学本科院校。新农合参合率提高到98.4%，38 所乡镇卫生院和112 个贫困村标准化卫生室建成使用，人口自然增长率控制在8‰以内。农村低保扩面由5.2% 提高到8.5%，新建乡镇敬老院12 所。开工建设保障性住房2.6万套，改造农村危旧房7000 户。实施扶贫项目1241 个，完成无房贫困户建房1023 户，新增脱贫人口17.4万人。实施移民后扶项目738 个，新增搬迁安置移民1815 人 侯卫东官场笔记。有效应对洪涝灾害，投入防汛抗灾及灾后重建资金7.8 亿元，灾民安置有序推进，房屋全倒户春节前可住进新居。积极发展文化广电体育事业，文艺创作获得国家级奖项11 项，广播电视村村通工程全面完工。成功承办湖南省纪念中国人民抗日战争胜利69 周年大会。组织举办全市第四届少数民族体育运动会，群众性文化活动和全民健身运动蓬勃开展。加强环境资源管理，启动大气污染防治四年行动计划，环境空气质量新标准自动监测系统建成使用，耕地保护实现占补平衡。深入开展民族团结进步创建活动，成功举办通道60 周年县庆，加强宗族场所教风建设，全市民族和谐、宗教和睦。加强矛盾排查调处，努力化解信访积案，特殊敏感时段进京非正常上访成为全省唯一的“零登记”市。建成社会治安视频监控探头1416 个，严厉打击涉黑涉恶势力及其“保护伞”、“黄赌毒”犯罪、多发性侵财犯罪和非法集资，全市社会治安综合治理考核排名全省第一。大力整改安全隐患，食品药品安全保障有力，安全生产形势平稳可控。支持工会、共青团、妇女儿童等各项事业发展，统计、档案、水文、气象、地方志、防震减灾、外事侨务、检验检疫、老龄老干、优抚安置、质量技术监督、住房公积金管理等工作取得新成效。

——严格落实政府作风建设新要求。深入开展党的群众路线教育实践活动，严格执行中央八项规定、省委九条规定，坚决反对“四风”，“三公”经费支出下降23%。建立市政府常务会、市长碰头会、市长办公会相衔接的决策和工作推进机制。出台《怀化城市规划区内房地产建设项目并联审批办法》、《市本级非税收入缓减免管理办法》等38 项管理制度。办理人大代表建议202 件、政协委员提案159 件，一批群众关注、事关全局的问题得到解决。市长热线和市长信箱受理群众咨询、建议和投诉2.9 万件，办复率99.3%。推进集中审批服务，办件受理57.5 万件，办结率99.9%。深入开展市直机关“万人评百人”活动和重点项目建设环境“百日清零”整治行动，加大行政监察、审计监督、网络问政和纠风工作力度，政风行风持续好转。明年任务尤其艰巨

明年是完成“十二五”规划的收官之年，也是我市“奋力创新业，建设新怀化”的开局之年。怀化经济必须构建一个契合时代特征、符合本市实际的新常态。这种新常态概括起来：就是以质量引领发展速度的新常态，更加注重经济质量和效益，并在保持不低于全省平均增速的基础上，努力增强提速的后劲和能力；就是以产业引领投资方向的新常态，推动投资更多地向产业集中，把优势特色产业逐步打造成市域经济的主要支撑，为怀化长远发展奠定更加坚实的产业基础；就是以改革引领经济活力的新常态，充分释放观念创新、制度创新、管理创新、科技创新的活力，在协同推进新型工业化、信息化、城镇化、农业现代化的深度融合中， 实现增长动力多元化； 就是以生态引领项目建设的新常态， 将生态理念融入城乡建设和生产生活， 促进产业发展生态化、生态建设产业化良性互动， 不断放大和转化生态优势； 就是以品质引领城市发展的新常态， 围绕打造居民更加舒适的生活空间， 持续推动城市综合提质改造， 提升城市引导消费、承载产业、服务居民的能力；就是以民生引领政府工作的新常态，把民生需求作为政府作决策、抓落实的基本取向， 坚持用民生成效检验政府的执政理念、行政能力、工作绩效， 真正建设人民满意的政府。

做好明年政府工作，总体要求是：全面贯彻党的十八大和十八届三中、四中全会精神以及中央、省委、市委经济工作会议精神，坚持稳中求进工作总基调，以全面建成小康社会为总揽，有力促进“三量齐升”，围绕建设“一个中心、四个怀化”，努力实现特色产业发展、中心城区综合提质改造、深化改革开放、文明创建“四个新突破”，突出创新创业，着力转型升级，加强民生保障，注重底线思维，切实提高经济发展质量和效益，推动经济平稳健康发展和社会和谐稳定。

主要预期目标是：生产总值增长8.5％左右；公共财政收入和地方财政收入分别增长10% 左右；全社会固定资产投资增长 15%；规模工业增加值增长12%；社会消费品零售总额增长12.5%；城乡居民收入分别增长10％和11%；城镇登记失业率控制在4.5% 以内；居民消费价格涨幅控制在2.5% 以下；人口自然增长率控制在9‰以内；全面完成省里下达的节能减排任务。

根据上述目标要求，明年重点抓好八个方面工作。

（一）坚定推进产业园区倍增计划。集聚资源， 集中精力， 奋战三年，实现全市产业园区主要经济指标翻一番。

—— 提升园区平台。加快完善园区基础条件， 积极争取省“135” 工程支持， 市财政安排1亿元资金支持基本建设，全年完成园区基础设施投资36 亿元，建设标准化厂房170 万平方米。改革园区管理体制，落实“一权两制一司”，将相应的行政审批事项下放到园区，逐步推行园政合一，建立相对独立的行政体制和财政体制， 组建独立的投融资公司。创新园区人才引进、薪酬激励等制度，优化园区运行机制。强化园区建设绩效评估，建立和完善科学的考核体系，实行重奖重罚，推进园区差异化、特色化、专业化发展。

—— 突出集聚发展。以怀化工业园为龙头，突破行政区划壁垒，规划建设怀黔千亿工业走廊。建立和完善利益共享机制， 鼓励各地将同类企业布局到专业园区，发展“飞地经济”。支持各产业园区培育壮大龙头企业，充分发挥产业链带动效应，积极推进同产业链企业分工合作、抱团发展。大力推广先进设备和高新技术，加强资源整合，实施品牌战略，改造整合以特色农产品加工为主的传统产业，加快培育电子信息、生物医药、新能源、新材料四大战略性新兴产业和商贸物流、特色农业两大优势产业，促进产业规模化、集群化发展。支持怀化工业园、怀化经开区打造千亿园区、国家级园区，推动各县市区工业集中区建设百亿园区。全市新增规模工业企业30 家以上。园区规模工业企业达到165 家、规模工业增加值增长30% 以上。

——培育骨干企业。全面落实稳定工业经济增长十条措施，立足现有资源，加强整合扶持，加大新兴产业项目引进、建设力度，对重点骨干企业实行“一企一策”帮扶， 协调煤、电、油、运、气等要素供应，帮助解决融资、招工和拓展市场等问题。依托骨干企业延伸产业链条，支持发展小微企业孵化基地，加强产业配套，促进小微企业竞相发展。

（二）强力推进中心城区提质改造。按照打造“五省边区生态中心城市”的战略定位，持续推进中心城区综合提质、深度提质，努力打造城市新形象。

—— 坚持以创新精神升华提质理念。遵循新型城镇化的要求，以“一本五化”的思路统筹中心城区综合提质改造。“一本”：就是以人为本。 “五化”：就是实行立体化改造，将主次干道与背街小巷、城市水系与地下管网、公共服务与城市空间、广告招牌与路边立面、亮化绿化与美化净化等等，一并纳入改造范围，通过系统谋划、统筹推进，实现功能和效益最大化；就是实行标准化作业，力求每一项工程、每一个细节让市民满意， 经得起检验；就是实行精细化管理，把城市管理延伸到每一个角落，切实解决公共空间脏、交通秩序乱、服务质量差、经营行为散等突出问题；就是实行生态化保障，加强城市水系、湿地、山体保护； 就是实行智能化支撑，着力打造智慧城管、智能交通、治安防控等数字化平台，积极探索智慧党务、智慧政务、智慧管理等现代手段，构筑面向广大市民的数字化公共服务体系，让广大市民享受智慧怀化带来的便利、快捷、实惠服务。

—— 坚持以科学态度把握提质规划。在城市格局的把握上，科学规划城市的对外形象，把东、南、西、北、东南、竹田等城市出入口打造成怀化的靓丽窗口；把迎丰路打造成为记忆怀化城市历史的东西轴线，把湖天大道打造成为展示怀化现代气息的南北轴线； 把迎丰路、人民路、鹤洲路合围的区域打造成为商业核心区，把迎丰路与湖天大道交汇点打造成为城市中央区，把高铁站周边打造成为城市形象展示区，把锦溪桥至太平桥段打造成为具有城市客厅性质的市民休闲区；把氵舞水河和太平溪打造成为人与自然和谐相处的城市生态景观区。在城市特色的定位上，把生态优势和人文优势深度结合起来，走具有怀化特色的城市化发展道路；在城市细节的处理上，每个片区的规划和设计，细化到每个楼栋的处理，每个楼栋的规划和设计，细化到每个楼面的处理，每个楼面的规划和设计，细化到每个窗户、每台挂机、每个门面的处理，让城市品质体现到城市的每一颗螺丝钉上，真正从细节上展现城市的高品质。

—— 坚持以开放思维拓展提质内涵。以“ 生活更美好、特色更鲜明、城乡更协调、机制更有效”为目标，按照先易后难、先急后缓、先近后远、先点后面和量力而行、逐年推进的原则，突出重点领域，把握关键环节， 深化提质内涵。成立中心城区提质改造总指挥部，并按分项目指挥部组织实施十大综合提质改造工程。道路建设与改造工程主要包括背街小巷改造，卢林北路和四方路建设，湖天大桥拓宽，城东高速公路延长线扩宽，迎丰路立交桥建设，红星北路、云集路、锦溪北路等断头路拉通，南环线接通和拓改，瑞丰路改造，刘塘路建设。太平溪综合治理工程主要包括太平溪源头治污，城东污水处理厂及截污干管铺设，太平桥廊桥建设，河道清淤。亮化工程主要包括城市主要道路亮化，城市重点区域亮化，城市桥梁亮化，城区重要节点亮化，城区路灯控制系统，城区风光带亮化。美化工程主要包括迎丰路、湖天大道功能性提质改造和临街建筑美化，高铁站站前广场、火车站站前广场、市委大院、市民服务中心周边建筑美化，太平溪两岸建筑美化。绿化工程主要包括迎丰路、湖天大道绿化提质，太平溪核心景观建设，迎丰公园提质改造，街头小游园建设，庭院绿化建设。智慧城市建设工程主要包括信息基础设施建设，智慧党务（政务）系统、智慧民生系统、智慧产业系统建设。公用事业设施建设工程主要包括城区供水管网建设，污水处理厂扩改建及提质提标改造，城区公交站场建设，城区垃圾中转站及公厕改造建设，二垃圾场后续配套，建筑垃圾消纳场建设，城区消防设施建设。农超市场建设工程主要包括城南农贸市场、芷江路农贸市场、天星坪农贸市场、锦绣五溪农贸市场、云箭农贸市场、舒家塘农贸市场建设，红星中路农贸市场、西站农贸市场和城东市场改扩建。棚改工程主要完成明年市本级城市棚户区改造任务11155 套。

同步启动国家卫生城市、省级文明城市创建，有效整治交通拥堵、渣土污染、噪声扰民、占道经营和乱搭乱建、乱贴乱画、乱吐乱扔、乱停乱摆等市民反映强烈的问题。

（三）扎实推进重点项目建设。

把项目建设作为经济工作的主要抓手，充分发挥投资对稳增长、调结构的拉动作用，全年固定资产投资突破1000 亿元。

——加大项目推进力度。抢抓武陵山片区区域发展与扶贫攻坚规划深入实施、“十三五”规划编制、国家重点支持七大类重大工程建设机遇，紧跟国家部委、省直厅局的项目布局和资金安排计划，对接省“511”重点投资计划，加强汇报衔接，确保更多项目进入国家、省支持范围。全力争取沅辰高速、石油储备库等重大项目落地。启动渝怀二线、怀芷高速建设和怀化铁路编组站搬迁。协调推进芷江机场改扩建和怀邵衡铁路、武靖高速公路等项目建设 金庸小说。推进国省干线改造升级，加快公路客运站场建设。

——提高招商引资实效。坚持领导带头，突出龙头企业招商、产业链条招商和特色园区招商，依托本地资源优势和企业优质资产，严格招商引资环保门槛，主动对接央企和国内500 强企业，加强对意向项目、签约项目的全程跟踪和服务，提高项目履约率、开工率、投产率。理顺招商体制，整合招商力量，优化招商机制，强化招商保障，全年引进市外境内资金480 亿元，增长25%。

——强化项目协调服务。实施项目审批流程再造，构建项目“绿色通道”，推动项目建设提速。强化银企对接，鼓励和引导各类社会资金参与重大项目建设，破解项目资金瓶颈。加大用地指标争取力度，深入开展闲置土地、违法用地清理整顿行动，化解项目用地制约。建立重大项目服务快速反应处理机制，坚决查处部门不作为、乱作为等问题，依法打击无理阻工、强买强卖等影响项目建设的行为。

（四）稳步推进强农惠农工作。

用现代工业化理念改造和提升传统农业，推进农业现代化和城乡一体化。

—— 大力发展现代特色农业。

按照标准化、市场化、专业化的要求，推进优势农产品基地建设，形成以粮油、蔬菜、水果、中药材、畜禽水产为重点的农业产业体系。稳定粮食播面490 万亩、总产180 万吨。

新增农业标准化核心示范园区20 万亩。培育新型农业经营主体，推进和规范农村土地流转，新增省级示范合作社10 家、市级以上龙头企业15 家。培育壮大农产品加工企业，扩大特色农产品精深加工，加快农产品物流中心建设，培育农产品加工物流体系，农产品加工销售收入增长20%。新增“三品一标”认证40 个，农机总动力增长12%。

——深入实施精准扶贫。扎实推进到户到人扶贫攻坚三年行动计划。积极开展产业扶贫，加快构建支撑扶贫的产业体系。推动水、电、路、气、房和环境整治“六到户”。

改善贫困地区办学条件，落实贫困学生补助政策，坚持分类指导，重点办好600 个扶贫示范点。全年实现脱贫17 万人以上。

——持续改善农村基础条件。加强水利设施建设，推进溆水大型灌区、长田湾中型灌区及红岩泵站群更新改造工程，除险加固水库水闸71 座，治理中小河流域7 处，实施五小水利工程3.3 万处，新建和加固堤防及护岸86 公里。抓好麻阳、辰溪、溆浦、芷江主城区和沅陵五强溪集镇防洪排涝工程。实施农村公路连通工程，加快通信、机耕道建设，升级改造农网6.79 万户，新建和改造农村低压线路3765 千米。抓好农业综合开发，建设高标准农田10 万亩。

（五）加快推进服务业提质转型。

对接高铁时代的到来，把服务业升级作为转方式、扩内需、强动力的重大举措，进一步优化经济结构。

——大力发展商贸物流业。坚持高标准规划、高品质建设，启动人民路、鹤洲路、迎丰路合围区域现代商业街建设，按照“经营实体+产品展示+ 仓储物流+ 网络交易”的新型市场运作模式，提质改造农产品、日用小商品、钢材、汽车、摩托车、服装布艺等传统市场，不断提高专业度。加快建设怀化国际汽车城等重点项目。规划建设一批仓储物流及配套设施。加快引进阿里巴巴、京东等战略型电商进驻怀化，构建“淘宝网——特色中国——怀化馆”电子商务平台，带动商贸物流业上档次、上水平。

——加快发展生态文化休闲旅游业。围绕建设全国生态文化休闲旅游胜地，精心打造芷江中国抗日战争胜利纪念地、通道国际侗族风情休闲区、古城古镇古村群落文化旅游区、沅水文化生态休闲带等四大旅游品牌。理顺旅游发展体制，创新旅游投入机制，实施通道旅游综合改革试点，努力建好全省文化旅游特色县。精心举办庆祝抗日战争胜利70 周年暨第五届芷江和平文化节。积极开辟沪昆高铁旅游走廊，主动对接张家界、凤凰、桂林等周边旅游市场。加快游客集散中心建设，全年旅游总收入增长15%。

——协调发展各类服务业。培育壮大信息科技、服务外包、健康养生、会展营销等现代新兴服务业，推进交通运输、住宿餐饮、家政服务等传统产业提质升级。深入开展省级金融生态良好城市创建工作，积极引进股份制商业银行和村镇银行，新增银行贷款100 亿元以上。规范和引导房地产市场健康发展，激活住房消费，稳定房地产市场。培育艺术雕刻、民族动漫、民族服饰、创意设计、演艺娱乐等特色文化产业，扶持壮大一批文化企业。

（六）深入推进改革开放和创新发展。坚持解放思想，大胆探索实践，着力增强经济发展的动力与活力。

——以更坚定的决心深化各项改革。抓好投融资体制改革，完善政府性投融资公司法人治理结构，引导融资担保业健康发展，健全风险防范和化解机制。推行PPP 融资模式，拓宽融资渠道。抓好城市建设与管理体制改革，抓好农村综合改革，溆浦农村土地确权登记颁证、麻阳农村产权制度、理顺市区财政体制，实现事权与财权匹配。完善国有资产管理机制，深化国有企业改革，抓好政府机构改革，深化行政管理体制改革，建立健全权力清单、负面清单、责任清单和乡镇便民服务中心“三张清单、一个中心”，推行行政审批“两集中、两到位”。创新市场监管机制，支持和引导非公有制经济健康发展。

——以更开阔的视野扩大对外开放。加快规划建设铁路口岸，积极申建海关办事机构，构筑开放平台，改善通关条件，促进外贸发展。加强与长株潭、长三角、珠三角及武陵山片区经济文化交流，深入推进以大（龙）新（晃）经济协作示范区为标志的省际区域合作，拓展厅市共建范围和深度。实施“走出去”战略，设立外贸综合服务试点企业，推进县( 市、区) 外派劳务服务站点建设，延伸市场空间。

——以更灵活的机制实施创新驱动。加强科技创新，积极开展“科技服务产业发展年”活动。围绕生物医药、绿色食品等重点产业，引导和帮助企业与高校、科研院所开展科技合作，加速科技成果转化和产业化。优化科技投入机制，强化知识产权导向，新增高新技术企业4 家，高新技术产品增加值达到57 亿元。

良好的生态环境是怀化的最大优势、最大财富、最大品牌，要像保护眼睛一样保护好这一方青山绿水和蓝天净土。

—— 不断提升生态文明理念。以抓好怀化“全国生态文明示范工程试点市”工作为载体，深入开展环保模范城、绿化模范城、生态乡镇及绿色学校、医院、小区、宾馆创建活动。加强生态文明制度建设，落实最严格的源头保护、损害赔偿、责任追究制度。

—— 重点治理环境突出问题。加大森林、河流、湿地、水库保护和修复力度，强化重点区域地质灾害防治。加强中心城区和县城饮用水源地保护，启动中心城区备用水源建设前期工作。推进“绿色通道、秀美村庄、美丽湿地”建设三年行动计划，治理裸露山地，完成高速公路沿线人工造林2.5 万亩、幼林抚育10.4 万亩、封山育林25 万亩、国省干道两旁绿化提质338 公里，建设秀美村庄示范村66 个。实施改水、改厨、改厕、改圈和垃圾集中处理“四改一集中”乡村环境综合治理工程，抓好靖州整县推进农村环境综合整治项目。推广农村沼气池、太阳能热水器、高效生物质灶等新能源。开展城区汽车尾气、燃煤锅炉、建筑噪声、扬尘污染专项治理，加强市区和各县城区空气质量自动监测，切实抓好大气污染防治。

—— 积极发展绿色低碳产业。加快产业转型升级，禁止新上高能耗、高污染项目。完成“ 十二五”节能减排目标任务。推进林纸、化工、水泥、工业硅等重点行业节能减排工程建设，实施废料、废渣、废液循环综合利用和废水、重金属污染治理。启动工业企业排污权交易工作，加强污水处理厂、垃圾处理场及工农业减排设施的运行监管。促进土地、林木、矿产等资源集约节约利用，提高利用效率。

（八）统筹推进各项社会事业。

以建设幸福怀化为主题，把发展重点向民生事业倾斜，努力加强社会建设，让广大人民群众共享发展成果。

——着力改善民生。新增城镇就业3.7 万人、农村劳动力转移就业5.2 万人。新建乡镇敬老院9 所。鼓励发展社会养老服务，扩大“空巢老人、留守儿童关爱服务中心”建设试点，健全农村留守人员关爱服务体系。新建集中供水工程372处，解决44 万农村人口饮水不安全问题。新建4.4 万套安居保障房。

——发展社会事业。坚持教育优先发展战略，深入实施义务教育均衡优质发展三年行动计划和城区教育第二个五年行动计划，建成义务教育合格学校80 所、农村公办幼儿园24 所、农村教师周转房4756 套，改造农村中学C 级危房11.5 万平方米，做好迎接全国义务教育发展基本均衡县市区的评估验收工作。抓好怀化职教基地建设，支持芷江师范争创怀化幼儿高等师范专科学校。加快建设湖南医药学院新校区。推进教育信息化建设，提升各类学校办学水平。完善基本药物制度，开展城乡居民大病保险，稳步推进公立医院改革，加快市三医院、市五医院、市妇保院、29 所乡镇卫生院、498 个村卫生室等项目建设。严厉整治违法生育，促进人口长期均衡发展。统筹推进公共文化服务体系建设，加强文化市场监管，促进文艺精品创作，实施直播卫星户户通等文化惠民工程。支持通道侗寨申遗，启动怀化侗族苗族文化保护区申报省级文化生态保护实验区工作。发展体育产业，促进体育消费，带动全民健身。加强国防动员、民兵预备役工作，抓好全国第七届双拥模范城验收迎检。优化住房公积金缴存、提取、贷款政策，充分发挥住房公积金的住房保障作用。统筹抓好宗教、老龄、监察、审计、统计、外事侨务、台湾事务工作，发展妇女儿童事业，促进地方志、档案、保密、气象、水文、防震减灾、检验检疫等各项事业协调发展。

——强化基层治理。以加强社区、乡村基层组织建设为基础，推进社会治理重心下移，将更多的人、财、物集中到乡村组织。积极推行简政放权，开展一站式便民服务。加强社区服务平台建设，推进社区网格化、信息化管理。保障乡村组织正常运转，保障基层干部正常待遇，鼓励和促进基层干部热爱基层、扎根基层、服务基层。

——维护安全稳定。深入推进法治怀化、平安怀化建设。强化维稳工作责任，落实重大事项社会稳定风险评估机制，抓好社会治安综合治理，加强人民调解和法律服务，有效预防和化解社会矛盾。坚持运用法治思维规范信访工作，推进诉访分离，实行依法逐级走访制度，及时就地解决群众合理诉求，加强流动人口和特殊人群管理服务，严密防范和打击各类违法犯罪活动。严格落实安全生产责任，坚决遏制重特大事故发生。推进食品药品监管体制改革，构建市县乡三级监管网络，突出重点问题专项治理，加大全程监管和风险防控力度。强化应急管理，完善自然灾害、事故灾难的监测预警和应急处置机制，提高救援和处置能力。政府自身建设责任重大

做好明年工作，必须把服务发展、服务企业、服务群众作为政府的第一职责，坚持以人民满意为标准，切实增强政府自身建设的针对性、系统性、实效性。

——强化公信力，打造公平公正的法治政府。注重用法治思维、法治方式推动工作、化解矛盾，增强行政行为的民主性、程序性、规则性。落实重大决策调查研究、专家咨询、风险评估、公示听证、合法性审查等制度，坚决贯彻落实市委决策部署，严格执行人大及其常委会的决议、决定，认真办理人大代表议案、建议和政协委员提案，自觉接受人大的法律监督、工作监督和政协的民主监督，坚决兑现政府各项承诺，努力建设人民群众可以信赖的诚信政府。

——强化担当力，打造善作善成的责任政府。坚持把担当精神贯穿治市理政各个环节，做到困难面前不绕道、矛盾面前不回避、压力面前不退缩。无论是解决事关全局的重大问题，还是解决事关民生的具体问题，都要增强阵地意识和主动精神，不计较个人得失和部门利益，以强烈的责任担当履行好自己的职责。特别是政府各级领导，要率先垂范，纠正推诿陋习，努力在实际工作中带头担当，在干事创业中升华境界。

——强化创新力，打造敢闯敢试的活力政府。坚持一切从实际出发，一切从人民利益出发，一切从发展大局出发，把改革创新作为推动政府工作的活力源泉。重点破解园区怎么建设、产业怎么培育、企业怎么扶持、城市怎么提质、资金怎么筹措、项目怎么推进、民生怎么改善等突出问题。

——强化执行力，打造立说立行的效能政府。宣战庸懒散，提振精气神。大力倡导雷厉风行、真抓实干的工作作风，确定的每项工作，要量化为指标，细化为项目，落实到部门，责任到人头，健全完善交办、催办、督办机制，实行全过程督促检查、全过程管理控制、全过程跟踪问效，确保工作推进有力度，督查有办法，落实有效果。严格问责机制，加大问责力度，真正让想干事的有机会、能干事的有舞台、干成事的有地位。

——强化自律力，打造清正清明的廉洁政府。坚持把思想教育作为打造廉洁政府的基础，正确看待手中权力和个人得失，强化干部自律，筑牢反腐倡廉的思想防线。坚持把制度建设作为打造廉洁政府的关键，健全管权管事管钱制度，完善行政运行机制。坚持把加强监督作为打造廉洁政府的保障，严厉查处各类违法违纪案件，坚决纠正损害人民群众利益的不正之风。尤其是在重点工程建设、财政资金使用、公共资源交易等领域，必须严格依法依规，把各级政府反腐败斗争进行到底，以廉政取信于民，以勤政造福于民。
